# Seeking maternal health care in rural Nigeria: through the lens of negofeminism

**DOI:** 10.1186/s12978-023-01647-3

**Published:** 2023-07-17

**Authors:** Ogochukwu Udenigwe, Friday E. Okonofua, Lorretta F. C. Ntoimo, Sanni Yaya

**Affiliations:** 1 School of International Development and Global Studies, Faculty of Social Sciences, University of Ottawa, Ottawa, Canada; 2 Women’s Health and Action Research Centre, KM 11 Lagos-Benin Expressway, Igue-Iyeha, Benin City, Edo State Nigeria; 3 Centre for Excellence in Reproductive Health Innovation, Benin City, Nigeria; 4 Federal University Oye-Ekiti, Km 3 Oye-Are Road, P. M. B. 373, Oye-Ekiti, Ekiti State Nigeria; 5 The George Institute for Global Health, Imperial College London, London, UK

**Keywords:** Maternal health, Nigeria, African feminism, Negofeminism

## Abstract

**Background:**

Feminist scholarship is acutely aware that health is not dependent on behavioural choices alone but on interlocking social determinants that affect people’s capacity to lead healthy lives. Women are situated within social structures that impact their health. but there is limited engagement with interpretive tools such as feminist theories that centre the realities of African women, particularly in the context of maternal health. It is imperative that women’s control over their reproductive health and autonomy in seeking care, particularly skilled maternal healthcare are understood within this context. This study seeks to examine pregnant women’s socio-cultural realities in a Nigerian context and in congruence with articulations of African feminism. Feminist scholarship acknowledges that women are situated within social structures that impact their health. Therefore, this paper seeks to examine pregnant women’s socio-cultural realities in a Nigerian context and in congruence with articulations of African feminism.

**Method:**

This is a cross-sectional qualitative study of a total of 64 participants: 39 women and 25 men in Ewato and Okpekpe communities, two Local Government Areas of Edo State in southern Nigeria. The study presents findings from eight sex-and-age desegregated focus group discussions. This study reports on emergent data related to women’s decision-making in accessing skilled maternal care. Data were transcribed and translated to English. Using the NVivo 1.6 software, data were coded and analyzed using a conventional approach to content analysis.

**Results:**

Findings describe ways in which women negotiate authority by ascribing the role of decision-maker to their men spouses while maintaining influence over their pregnancy healthcare decisions and actions. Negofeminism’s concepts of alliance, community and connectedness were highlighted through men’s constructive involvement in maternal health. Furthermore, women were shown to maneuver patriarchal norms to gain control of their healthcare decisions.

**Conclusion:**

This study offers a different narrative from the dominant view of non-Western women, specifically African women, as oppressed passive victims who are ineffectual in taking charge of their health. From the perspective of negofeminism, women navigate patriarchal environments to yield the best possible maternal health outcomes. The current study can be useful in informing policy and programming that acknowledges women’s social embeddedness.

## Introduction

Feminist scholarship in bioethics generally strives to advance health and well-being across diverse cultures and traditions by bringing attention to major injustices that override local boundaries [1]. Within this scholarship, there is an understanding of the manifestation of power hierarchies in the distribution of health resources at the local, national and international scale [2]. Feminist scholarship is acutely aware that health is not dependent on behavioural choices alone but on interlocking social determinants that affect people’s capacity to lead healthy lives. In a related vein, feminists acknowledge that women are situated within social structures that impact their health. For instance, studies have shown that unequal power dynamics between men and women caused women to have limited control over their sexual and reproductive lives thereby limiting their timely access to care [3]. It is imperative that women’s control over their reproductive health and autonomy in seeking care, particularly skilled maternal healthcare are understood within this context. This study seeks to examine pregnant women’s socio-cultural realities in a Nigerian context and in congruence with articulations of African feminism.

### The state of maternal health in Nigeria

This study focuses on Nigeria where the global burden of maternal mortality weighs heavily. Nigeria, Africa’s most populous country, accounted for nearly 20% of all global maternal deaths [4, 5]. Specifically, Nigeria records 556 pregnancy-related deaths per 100,000 live births. Nigeria’s Demographic Health Survey estimates women’s lifetime risk of maternal death to be 1 in 34, meaning that 1 in 34 women will have a death related to maternal causes [5]. Under the third Sustainable Development Goal, countries have united to reduce the global maternal mortality rate to less than 70 deaths per 100,000 live births by 2030 [6, 7]. However, current trends indicate that Nigeria risks falling short of this target. Most maternal deaths in Nigeria result from obstetric complications that could be prevented with timely access to skilled maternal services. The three-delay model is a prominent framework that describes the sequential and interrelated phases of delays that lead to maternal deaths and morbidity. Women’s limited autonomy in decision-making can cause delays in seeking care (phase 1 delay), distance from healthcare facilities can cause delays in reaching adequate healthcare (phase 2 delay), and inadequate medical assistance can cause delays in receiving adequate care [8, 9]. Most maternal deaths in Nigeria have been attributed to a combination of these factors. An extension of the three-delay model reinforces preventive action that averts these delays [9, 10]. The potential for preventive action on maternal health lies heavily on an understanding of women’s general status in the context of her community. For instance, studies have shown that women’s partners, family members such as mothers or grandmothers, and community members are key players in maternal health as they influence women’s healthcare-seeking behaviour [11].

A fourth delay proposed by MacDonald et al. attributes poor maternal health outcomes to delays in community action or lack of collective responsibility [9, 10]. Maternal health is impacted by a community’s capacity to self-identify norms and expectations around health service use, identify and harness resources available to them, and mobilize efforts to prevent and reduce maternal health. This fourth delay acknowledges women’s interconnectedness and the relevance of their relationships both at familial and community levels to maternal health. Delays arise when a community is not sufficiently knowledgeable or aware of maternal health, or where there is an absence of collective responsibility to drive political action on issues pertaining to maternal health [9]. Community-based research studies bring to light the role of community efforts to enhance healthcare services [10, 12–14]. These efforts include the provision of land and labour to build healthcare facilities, collective action for demanding accountability from healthcare services, and mobilized efforts towards improved community infrastructure such as better roads and transportation services. In exploring the role of women’s community in maternal health, the following section draws upon a wider concept of personhood that extends beyond the individual.

### Autonomy in the context of maternal health

Studies across Africa have noted that the lack of autonomy as it relates to decision-making in maternal health has adverse effects on women [15–17]. However, concepts such as autonomy are highly contested in the literature and bring to the fore arguments about its definition, coherence and subsequent application in different contexts [18, 19]. For instance, feminists have spoken about the rigid individualism embodied by the concept of autonomy. The overemphasis on masculinist ideologies of domination and personal control neglects relational values [1]. Acknowledging other realities such as the relevance of social networks to maternal wellness is important to drive appropriate action toward reducing maternal mortality. It is for this reason that feminists have sought to revise the concept of the autonomous person and the autonomous decision-maker to include relational autonomy. The notion of the autonomous individual- independent, rational and self-interested-is strongly influenced by Kantian ideas of rational agents [20, 21]. This rational choice lens promises a measurable and predictable approach to understanding personhood and human action, however, its flawed conceptualization of people as inherently independent and self-interested ignores collective responsibility and shared social practice evidenced in different cultural settings.

Relational autonomy balances the argument of the rational and autonomous person with arguments of the realities of a person’s social embeddedness [21, 22]. Some feminist scholars argue that being able to reflect on choices and their consequences affords a person some sort of autonomy. Stoljar asserts that “preferences for relationships of care and dependency such as those within marriage or other family structures can be just as autonomous as preferences for self-reliance or relative social isolation; preferences for cultural and religious norms into which agents are born can be just as autonomous as preferences to repudiate these norms” p. 7 [23]. This argument provides a necessary framing of agency for situations that have previously been interpreted as oppressive and offers up a different understanding of choices and forms of agency, particularly in a non-Western context. For instance, the emphasis on women’s sole decision-making for maternal healthcare often precludes considerations of couples’ joint decision-making as an important determinant of maternal health [18].

In a similar vein, African feminism challenges the foreign gaze imposed by Western feminist scholarship that subsequently dictates narratives of feminism in an African context [24, 25]. African women, often seen as the ‘other’ are depicted as subordinated, dominated and passive subjects of patriarchal structures in their society. Western feminist discourse of autonomy and empowerment in maternal health is also not immune to ideologies that privilege individual freedom, choice and personal responsibility without the necessary critique of the systems that constrain women [26]. In describing Western feminism’s scrutiny of human agency, Nnaemeka posits that “Western feminism is caught up in its ambivalence: fighting for inclusion it installs exclusions; advocating change it resists change; laying claims to movement, it resists moving” p. 363. This serves as a reminder that while feminism as understood and practiced in a Western context can offer important perspectives for maternal health, it can be lopsided in theorizing women’s realities across non-Western contexts and is therefore not wholly applicable in an African context. Therefore, to understand women’s lives in an African context, one must refer to African feminist theories that centre the stories and histories of African women within the cultural and philosophical spaces they occupy.

### Maternal health through the lens of negofeminism

The authors agree that in theorizing women’s authority in maternal healthcare across cultures, one must interrogate the source of the theory and its positionality through which a social, intellectual and political stance is legitimized. A glaring risk of failing to localize theories for subjects of research is the danger of homogenizing their lives [27]. The authors agree that the location of African women as knowledge producers and subjects of knowledge production is imperative to avoid the pitfall of homogenizing women’s experiences in line with ‘victimhood’[25]. Not only does this trope of victimhood negate women’s agency, but it also perpetuates gender inequities. Discourses on feminism in Africa reflect the differences in religion, ethnicity and regions of Africa but they share a common theme of complementarity of genders, equity and justice for both women and men. African feminists assert that aspects of Western feminism engagement such as the call to ‘disrupt’, ‘challenge’ or ‘blow apart’ are antithetical to feminist engagement in an African context where the approach is to ‘collaborate’ and ‘negotiate’ [24, 28–30].

Nigerian feminist scholar, Obioma Nnaemeka describes feminism in an African context as feminism of negotiation or negofeminism [24, 30]. Negofeminsim strives for gender equality through negotiations and compromise. It involves situational ‘give and take’ exchanges within the dominant culture in lieu of confrontational exchanges. In a maternal health context, the dominant culture can be driven by factors such as patriarchal norms around decision-making authority or men’s obligations during pregnancy. In Nnaemeka’s words, African women do feminism for themselves but also as acts of altruism. Importantly, African women understand that context is important for tackling patriarchy, therefore negofeminism knows when/where/how to negotiate around patriarchy and when/where/how to overthrow patriarchy. Snail-Sense feminism, a similar African feminist theory proposed by Akachi Adimora-Ezeigbo adopts the metaphor of a snail to indicate the process through which women navigate oppressive conditions [28]. The theory alleges that much like the snail, women survive patriarchal conditions by dealing with men slowly and retracting in difficult situations. Similar to negofeminism, snail-sense feminism is underlined by principles of negotiation and cooperation but has been problematized for its depiction of cowardice or subjugation as a means to address gender inequality [28]. In a maternal health context, this theory would encourage women’s negotiation around seeking maternal healthcare but would also encourage their quiet recline if men disapprove of their attempts at negotiation. In contrast to the snail approach of retracting, negofeminism recognises the need to “challenge” and “detonate” through dialogue and negotiation even in unfavourable conditions. These nuances make negofeminim a more suitable lens through which to explore maternal health in this paper.

Negofeminsim in practice is pervasive in an African context. For instance, while aiming to achieve gender equity, the Maendeleo Ya Wanawake Organization, a women’s non-governmental organization in Kenya successfully deployed negofeminsim in their campaign against female genital mutilation [30]. Women recognized the cultural significance of cutting in the broader context of a central rite of passage that ushers girls into womanhood. Their campaign successfully eliminated the harmful practice of cutting while retaining the cultural significance of the ceremony [30].

Against this backdrop, a gaping issue is the limited engagement with interpretive tools such as feminist theories that centre the realities of African women, particularly in the context of maternal health. Therefore, this paper uses African feminism, specifically negofeminism as a theoretical underpinning for exploring maternal healthcare decision-making and wellness in rural Nigeria. Through conversations with men and women on maternal healthcare seeking and maternal health wellness, this paper examines how discourses underlying negofeminism are tied to maternal health.

## Methods

### Study design

This study forms part of a larger study by the Women Health Action Research Centre (WHARC), a research based organisation aimed at improving maternal health in rural Edo, Nigeria. This is a cross-sectional qualitative study of women and men involved in the larger study and presents findings from focus group discussions (FGDs) with them. The study takes a subjectivist inductive approach which follows assumptions of socially and experientially constructed realities through an individual (or group)’s interpretation and understanding of reality [31]. The researchers have a responsibility to acknowledge these realities and explore meanings constructed by individuals. Specifically, this study uses a theory-informing inductive data analysis study design, meaning that this study employs theory, in this case, negofeminism, as an interpretive tool but not as a basis for conducting and designing the study. By acknowledging that reality is subjective and varies from person to person and by centering the participants as holders of knowledge, this study draws on constructivist ontology and epistemology [32].

### Research setting

The study is set in Ewato and Okpekpe communities, located in two predominantly rural Local Government Areas of Edo State, in southern Nigeria. Edo State is one of Nigeria’s 36 states, the majority of Edo’s population of over 4 million people reside in rural areas. The two communities comprise 20 villages and hamlets. Each of the two communities have two primary healthcare centres (PHCs) making a total of four PHCs covering the 20 villages. These communities were chosen because preliminary baseline assessments revealed high maternal mortality rates and low use of primary healthcare facilities. Furthermore, these communities are located in rural areas where there are no secondary or tertiary health facilities in the vicinity. PHCs serve as their nearest healthcare facilities.

### Participant sampling and recruitment

There were ongoing research or intervention activities in the study sites, therefore we anticipated that most potential participants would already have experience with the WHARC team. Participants were purposefully chosen from WHARC’s database containing names and contact information of women participating in maternal health interventions. Women consented to have their contact information collected and stored on the database. Their contact information was only made available for research. Two research assistants conducted data collection. The research assistants, one man and one woman, each had a bachelor’s degree or higher level of education and are experienced in qualitative method data collection. They made telephone calls to women to solicit their participation and followed a telephone script for participant recruitment developed by the lead author (OU). Eligible participants were women between the ages of 15 and 45 years old who participated in maternal health intervention programs during their most recent pregnancy. Men spouses were contacted through snowball sampling whereby women invited their spouses through word of mouth to participate in the study. Research assistants followed up with men and introduced the study and solicited their consent to participate. All women who were approached agreed to participate in the study but some of the men declined to participate.

### Data collection and procedure

The two research assistants conducted a total of eight focus group discussions with a total of 64 participants across the two communities between September 2021 and January 2022. Following a recommendation by Fusch and Ness, each group consisted of 6–10 participants. Groups were small enough for members to talk and share their opinions yet large enough to create a diverse group [33]. Focus group discussions occurred at community squares. Research assistants and participants adhered to COVID-19 protective measures during the study which included wearing masks and social distancing. Sanitizers were also made available to participants. Each participant received 3000 Naira ($10CAD) to cover transportation and refreshment expenses. Prior to participating in this study, participants provided their informed consent by signing a consent form. Research assistants facilitated FGDs in English or Pidgin English language.

Questions were translated verbally into the local dialect when necessary. Each FGD was digitally recorded, and researchers took notes during the discussions. To allow for sex-desegregated data collection, FGDs were held separately for men and women. This was important to encourage open discussions of private experiences and minimize undesirable consequences such as spousal confrontation or abuse that may threaten the participants’ or their family’s stability [34]. Women and men groups were further segmented by age as this helps to minimize the effects of age hierarchy as dictated by cultural norms which may force younger participants to withhold their opinions [34]. Discussions were simultaneously translated and transcribed to English and were reviewed and discussed by the lead author to ensure a shared concept of key terms. Other coauthors with proficiencies in both languages re-examined the translated transcripts and screened them for errors. Any identifying information for participants was altered to protect their privacy, they were referred to simply as a man or woman participant instead. Participants’ responses were either transcribed verbatim if they responded in English or translated if they responded in Pidgin English. Literal translation (word-by-word) was used to preserve participants’ responses and provide readers with an understanding of the mentality of the participants [35].

### Research instrument

The lead author designed qualitative FGD guides to capture contextual information related to the topic and elicit detailed discussions from women and their spouses regarding their opinions and lived experiences as it relates to seeking maternal healthcare and maternal health in general. Questions were carefully crafted to include neutral, non-biased, and non-leading questions to avoid influencing participant responses. FGD guides were modified where appropriate to suit the local language, literacy levels and cultural interpretations. The current study reports on findings specific to maternal healthcare decision-making and maternal wellness in general. A sample of issues discussed with participants include:

1. The first person a woman consults for general pregnancy care, and more specifically during pregnancy complications.
2. The major decision-maker (s) for seeking maternal healthcare.
3. Lived experiences of men’s involvement in maternal and child health from the perspectives of fathers.

### Data analysis

The transcripts were exported to NVivo 1.6 and OU led the data coding process. This study used a conventional approach to content analysis to explore responses from FDGs. In this process, the coder was immersed in the data and allowed new insights to emerge. There was no reliance on a pre-existing category, instead, inductively, categories flowed from the data [36]. Codes captured data relevant to the primary questions of this study as well as emergent themes that arose from the initial review. In an iterative process, the coder defined codes that appeared meaningful. Although the codes emanated from the data, the coder acknowledges the influence of their background and experiences in defining codes. Upon identifying and defining codes, representative quotations from the transcript were assigned to different codes. Codes were reviewed and overlapping codes were further organized into categories. Themes were subsequently generated from categories that reflected a level of pattern in response or meaning. Salient themes were identified as those that reflected experiences around maternal healthcare decision-making and wellness. Approach to data analysis used a theory-informing inductive data analysis study design, meaning that theory, in this case, negofeminism, was used as an interpretive tool for emerging data but not as a basis for conducting and designing the study [31].

### Trustworthiness

The authors adopted various strategies to ensure trustworthiness in this qualitative study following suggestions by Shenton [37]; FGDs were structured to allow for iterative questioning including the use of probes to elicit detailed responses, questions were rephrased to participants when necessary. The authors conducted member checks after data collection and data analysis was reviewed by SY to ensure accuracy of the data [37]. All authors reached a consensus on emerging themes and the manuscript provides thick descriptions of the phenomenon of interest.

## Results

The total of 64 participants comprised 25 men and 39 women. Participants ranged from 6 to 10 per focus group discussion. The median age for women was 26 years old and 45 years old for men. The predominant level of education in these rural communities is primary education and the conventional occupation is farming. Please see Table 1 for a breakdown of participants in focus groups.

**Table 1:** Study participants

Participants	Contact type	Number of participants	Age range (median) in years
1. Ewatto women 1	FGD	6	34–39 (36)
2. Ewatto women 2	FGD	8	25–30 (27)
3. Okpekpe women 1	FGD	8	45–52 (45)
4. Okpekpe women 2	FGD	10	40–47 (45)
5. Okpekpe women 3	FGD	7	52–57 (54)
6. Ewatto Men	FGD	8	25–28 (26)
7. Okpekpe men 1	FGD	8	20–25 (23)
8. Okpekpe Men 2	FGD	9	24–29 (26)

The following section examines how discourses underlying negofeminism are tied to maternal healthcare-seeking and maternal health in general. Men’s and women’s responses are categorized as negotiation, collaboration and maneuvering, all basic tenets of negofeminism [24]. This approach is not intended to essentialize hegemonic masculinity that establishes men’s dominance over women thereby severely limiting their reproductive rights [38]. Instead, this section offers a different narrative from the dominant view of non-Western women, specifically African women, as oppressed passive victims who are ineffectual in taking charge of their health [25]. From the perspective of negofeminism, findings show how women navigate patriarchal environments to yield the best possible maternal health outcomes.

### Negotiating authority

Decision-making in maternal healthcare is an important point of departure as it situates women and men in the reality of their interdependence through social relationships and how this interdependence impacts maternal health goals. Responses from participants indicate that decision-making authority was predicated on gender roles and norms. Gender incorporates one’s biological sex, sense of self and socially constructed characteristics and is highly influenced by roles, rights and obligations in a given society. A common theme was the evidence of a descriptive role of men and women at the household level. Descriptive in the sense that it highlights beliefs of what men and women should typically do even if those expectations are not carried out in reality. Evidently, men were considered the decision-makers at the household level. Participants opined that a woman’s spouse should be the first to know of her pregnancy once confirmed.

*“When a woman finds out she is pregnant, the first thing she does is to thank God, then she tells her husband.” (Woman participant, Ewatto)*

*“Number one, she has to first give the report to her husband. She then goes to the nurses to confirm whether signs of pregnancy are true.” (Man participant, Ewatto)*

Upon receiving the confirmation of a pregnancy, men were deemed the decision-makers in seeking maternal healthcare. This position was verbalized even when there was evidence of joint decision-making between men and women. Men’s views of their decision-making authority were tied to their role and duty as “fathers of the house”, a role the men collectively took pride in and indicated their responsibility towards their spouses during pregnancy. A salient response was how it was “normal and common” for the man to perform his responsibilities as the father of the house.

*‘Because you cannot just go without the husband’s decision. We as fathers of the house, it is our responsibility to ensure that everything takes place properly.” (Man participant, Okpekpe)*

*“Normally, if you and your woman stay together, you are supposed to know each other’s problems and share. So she is supposed to say my husband, I am not feeling well and he will say Ok, go and see the doctor.” (Man participant, Okpekpe)*

Similarly, women ascribed the role of decision-makers to men but would also narrate instances where they influenced the decision to seek medical care. For instance, it was not uncommon to hear participants say that after confirming their pregnancy and informing their spouses of the pregnancy, women *tell* their spouses to take them to the health facilities. The use of language to influence decisions on access to maternal healthcare can be thought of as a form of negotiation on the part of women. First, she recognizes the patriarchal environment and ascribes the decision-making authority to men but is also actively enacting her agency within that environment. Furthermore, men reported that their spouses would often make the sole decision on seeking skilled care in their absence. Several participants reported that their spouses were able to seek skilled care if they (the men) were absent from the home.

*“In the aspect of care, I will tell my husband, so he will decide. After my husband knows, I will go to the hospital to tell the doctor so he can tell me what to do.” (Woman participant, Okpekpe)*

*“Sometimes, your wife will tell you “take me to go and see nurse’. When I am not around, she can go see the doctor on her own. It is a normal thing in our community.” (Man participant, Okpekpe)*

It is noteworthy that regardless of who made the final decision, men and women in this study unanimously acknowledged the importance of seeking skilled maternal healthcare, particularly during obstetric complications. In one instance, a woman mentioned in order of importance, consulting a nurse first at the onset of an obstetric complication and secondly, consulting her mother. Men also stated that pregnant women needed to have frequent hospital visits to ensure the baby’s survival. This indicates that men and women were generally knowledgeable about the importance of seeking skilled care during pregnancy.

### Collective responsibility for maternal health

African feminism conceives men and women as critical halves of the human whole, men are therefore seen as equally important in improving the lives of women. This section describes men’s involvement in maternal health and shares men’s opinions of the constructive role they play in maternal health. It is important to highlight, however, that men’s role in maternal health is nuanced and can vary based on location and contexts. In this case, men displayed positive and collaborative roles in ensuring women’s optimal health during pregnancy.

Men indicated their familial obligation to their pregnant spouses and their perceived roles as fathers. Salient themes around men’s responsibilities were of financial, supportive and domestic responsibilities. Men’s financial obligations during pregnancy included ensuring financial resources were available to cover the cost of maternal healthcare visits such as antenatal care, childbirth and other non-routine services. They admitted that fulfilling their financial obligations was sometimes a challenge but deemed it their responsibility, nonetheless. Another common theme pertaining to men’s responsibilities was ensuring that women reached healthcare facilities. They coordinated transportation for women or accompanied them to healthcare facilities even when it was not convenient to do so. Some men noted they had to *abandon* other tasks and accompany their spouses. Additionally, they reported that they took on domestic responsibilities around the house during their spouse’s pregnancy.

*“As a father, as the head of the family, it is our own responsibility to ensure that our women get to the place [health facility] and return when it is time for childbirth. The woman has to go for scanning and the father has to be aware of the date and time so he can safeguard her transportation to and from. On the day of childbirth, he has to shoulder the responsibility of ensuring her transportation to the nearest health facility to have the baby. I have to be with her when labour starts. The husband is the head of the household and has those responsibilities.” (Man participant, Okpekpe)*

*“The normal thing in this community is that he [the man] is supposed to give his woman good food, treat her well and take good care of her because of the baby. He should always make sure she takes her medication. Then he should pray that God will be with her before she delivers the baby. After the delivery, he will still continue to take care of her.” (Man participant, Okpekep)*

*“Every week, I have to take her to attend clinic regularly, every Tuesday or so, I will make sure I give my wife money too.” (Man participant, Ewatto)*

Their notions of responsibility and collectivity are key to conversations about maternal health.

It can be argued that in ascribing decision-making authority to men, women benefit from men’s duty and responsibility to be providers. Evidently, women did not have the wherewithal to cover their maternal healthcare costs themselves. One woman affirmed the high cost of maternal healthcare services and the inability to pay for them on her own. This evokes the notion of “give and take” associated with negofeminism. This exchange, however, does not necessarily involve men relinquishing the power that patriarchy affords them such as power over financial resources or power over decision making. What negofeminism offers is a lens to view how women find ways to go around patriarchal contexts to ensure they have access to skilled care and remain healthy during pregnancy. It is important to note that this study did not explore women’s economic vulnerabilities because previous studies by the authors in the same communities have already reported on the gender dynamics impacting women’s economic power [39].

### Maneuvering control

Findings revealed women’s agency in accessing and using maternal health services offered in their communities unbeknownst to their men spouses. During focus group discussions with men in one of the Okpepe communities, men had reportedly never heard about services offered for maternal care. However, group discussions with women from the same community confirmed that not only were women aware of these services, they were also using them. Men reasoned that their spouses were uncomfortable telling them about maternal healthcare services or they felt that the men would not be interested in knowing about them.

*“Like sometimes women will attend some of those programs, maybe, some of them may feel reluctant to tell their husbands. My wife didn’t tell me anything about it. Maybe my wife received it, but you know sometimes everything that women do may not be what men do.” (Man participant, Okpekpe)*

While it is not clear the context within which women decided to withhold information from their spouses, women can be argued to have displayed their agency in taking the necessary precautions to safeguard their health during pregnancy. Furthermore, some women indicated that they were solely responsible for deciding when and where to seek maternal healthcare.

*“I am the one with the pregnancy, so I am the one to decide to go to the hospital, but then I will tell my husband.” (Woman participant, Ewatto)*

## Discussion

This paper examined maternal healthcare seeking and wellness in a rural Nigerian context by exploring how negofeminism is deployed in maternal healthcare seeking. By using a theory that is closest aligned with the realities of participants, the negofeminist position centres the African woman’s stories and histories and highlights ways in which she negotiates with and subverts patriarchy. Findings show how women navigate patriarchal environments through negotiation, collaboration and maneuvering to yield the best possible maternal health outcomes.

Negofeminism posits that each gender (along the male/female binary) constitutes the critical half that makes the human whole. Furthermore, a common notion in Southern Nigeria where this study is located, is that the maintenance of harmonious gender relationships between men and women is critical to everyone’s well-being [24, 29]. For instance, women have been shown to collectively hold men responsible for injustices that impacted their lives thus indicating a recognition of the role of men in ensuring women’s well-being. Findings indicate that women ascribed the role of decision-maker to their spouses in recognition of men’s role and responsibility in ensuring maternal wellbeing. Women described their spouses as the decision-makers even in instances of joint decision-making. Implications of this finding highlight the importance of broadening the focus on women’s sole decision-making autonomy and examining the role of joint decision-making in enhancing access to skilled maternal care. Studies across different African contexts have reported on women’s sole decision-making as a proxy for their autonomy or power with the argument that women lack autonomy if they cite their male partners as decision-makers in maternal healthcare [40, 41]. However, the focus on sole decision-making can obscure other ways in which women participate and determine their own fate.

Similar to findings from the current study, a Nigeria-based study makes a case for joint decision making as an acceptable autonomous process when it represents women’s values and preferences in certain societies. The study affirms that couples’ joint decision-making can hold benefits for maternal health outcomes more than decisions made by only women [18]. Other studies highlight men’s role in maternal healthcare such as their financial and emotional support to navigate barriers related to seeking maternal healthcare. Decision-making with men across low and middle-income countries was shown to increase antenatal attendance, maternal nutrition, skilled birth attendance, and postpartum care [42].

In addition to drawing attention to men’s role in maternal care, this study also explores and challenges the discursive legacies of Western values that can make their way into maternal health research and practice on a global scale [43]. African feminists interrogate the discursive legacy of colonialism manifested in practice by juxtaposing the terminology around ‘culture’ in a Western vs. a non-Western context. Certain acts such as rape or spousal murder are described as ‘culture’ when they occur in a non-Western context whereas those acts are termed ‘acts of violence’ not culture when they occur in a Western context [24]. These narratives reiterate the assumed superiority of western lifestyles and informs interventions that implicitly construct culture in a non-Western context as a barrier to maternal healthcare. This simplistic view of culture that excludes the knowledge and complex realities of intended recipients results in well-intentioned maternal health interventions going awry [29, 44].

African feminism highlights the notion of culture in an African sense as a dynamic and evolving hybrid of new totalities from different historic and geographic contexts [24]. It shows how women work with men to achieve set goals of maternal health and wellness. Importantly, it highlights the importance of an engaged community to support maternal health. African feminists posit that in an African setting, individuals are motivated by community validation and will act in ways to earn or retain their community’s validation [29]. In the current study, men’s narratives of ‘normal’ and ‘common’ practices of expectant fathers in their communities is an ideal space to explore notions of rigid gender norms and roles. Men reported that they undertook domestic responsibilities such as cooking, washing clothes and child-rearing, to ensure a healthy pregnancy for their spouses. These activities were considered normal for expectant fathers in their communities. The deviation from rigid gender roles could be attributed to communities normalizing these responsibilities and cultural imperatives that prioritize maternal health and wellbeing at the community level which reinforces men’s positive behaviour at the household level. In line with African feminists’ definition of culture, findings from this study speak to gender norms as being malleable and not generalizable in an African context.

This argument is consistent with findings from a similar study in Sierra Leone that affirms men’s high level of involvement in maternal health despite the dominant “absent male involvement” rhetoric in the global health literature. Expectant fathers were involved in gendered labour and caregiving activities [45]. Men’s involvement also extended to financial responsibility which is an important role in the context of underfunded health systems and lack of access to financial resources for women [12, 46]. Another study, however, reported a contrary finding. Expectant fathers in Tanzania were less likely to undertake gendered labour but were heavily involved in facilitating their pregnant spouse’s access to health facilities by supporting their partners financially and securing necessary items for labour and delivery [47]. Similarly, findings from Northern Nigeria indicate that while expectant fathers were key decision-makers in maternal health and providers in a financial sense, they rarely deviated from strongly held traditional gender norms [48].

Additionally, findings from this study revealed women’s resistance to men’s involvement in their pregnancy. Some men were not aware of their spouse’s use of maternal services during pregnancy. Some women reported seeking maternal health care on their own terms. These findings defy the positioning of women in an African context as “passive and oppressed”. By maneuvering norms that would have conditioned care seeking on a man’s authority, women are indicating their control over their lives and maternal healthcare decisions. This finding was corroborated by a similar study in Ghana where women resisted men’s involvement in their pregnancy [49]. Women stated that the presence of a man limited the care and interaction with their healthcare providers. Women also felt that handling their pregnancy on their own gave them a sense of pride, control and self-actualization. This finding also closely aligns with Nnaemeka (2004)’s stance that feminism is what African women do for *themselves* as well as for others. The emphasis is intentional as it highlights that feminism for African women is not always an altruistic act. African feminism does not appear to support the singular imagery of the selfless African woman but seemingly acknowledges the complexities of African women whose ideologies can go beyond that of negotiation and ways in which they consciously resist.

### Strengths and limitations

The strength of this study lies in its use of open-ended qualitative research which allowed the emergence of key issues in maternal health. The use of African feminism as an interpretive tool provided insights into the communities’ realities that have been diminished through the lens of dominant but not localized theories. The study draws on diverse perspectives and experiences of men and women as it allowed for an exploration of latent themes, for instance, women’s influence over their healthcare even with gender expectations that hold sway in communities.

While this study offers important perspectives on maternal health, the reported findings should be interpreted in light of a number of limitations. The degree to which probing was employed as a technique by the research assistants may have influenced the responses generated by participants. However, it is noteworthy that the use of local research assistants who could converse in the local languages undoubtedly impacted the dynamics between participants and researchers and improved rapport. Another important limitation is that by design, participants were purposefully selected therefore does not represent the views and realities of all men and women in rural areas of Nigeria. The realities of men and women will differ across Nigeria’s various geopolitical zones. The study aimed to get information from men and women who participated in the parent study, therefore, perspectives of fathers and mothers who did not participate in the parent study are not included in this study. Finally, this study could have benefited from deeper integration of an Afrocentric paradigm whereby the use of Afrocentric theory/theories would have informed every stage of the research process including the development of research questions, methodological choices, data collection, data analysis and conclusion.

### Policy recommendations and future research

The implications of this study are important for policy development. This study broadens the understanding of maternal healthcare beyond individual (women’s) responsibility but extends that responsibility to the community and nation at large. The responsibilization of mothers in maternal healthcare has been noted as a strategy to improve maternal health in African countries [43]. Strongly associated with neoliberal ethics that privileges individual choice and personal responsibility, responsibilization refers to the process of rendering women responsible for their health outcomes without the necessary critique of the systems that constrain women [26, 50].

By situating maternal health as an outcome of individual behaviour and ignoring African women’s sociopolitical realities, this narrative risks undermining interventions that aim to improve maternal health. Therefore, the current study can be useful in informing policy and programming that acknowledges women’s social embeddedness with their communities.

Moreover, this study highlights the importance of involving men and women in the design and implementation of maternal health interventions in a way that accounts for their gendered roles and gender dynamics. Men’s involvement in an African context should also be recognized and supported. Interventions aiming to involve men in maternal health could build on men’s already existing constructive roles while providing opportunities to deepen their involvement. This will involve multisectoral strategies to address individual, structural and systemic barriers to men’s involvement in maternal health [42]. Joint decision-making as a strategy to improving maternal health was a common theme in this paper. This concept shows promise particularly in contexts where care-seeking reflects a communal nature of decision-making in communities. Efforts to encourage women’s use of skilled maternal healthcare can move beyond a focus on the individual (women) and increase community responsibility for maternal healthcare [40]. Furthermore, studies indicate that couple’s joint decision-making for maternal healthcare is relatively under researched as there tends to be a focus on shared medical decision-making between patients and healthcare providers [18]. There is a need for more research to expand understanding on joint decision-making and evaluate the presence of power imbalances that could impact couple’s joint decision-making.

This study can also inform community engagement strategies through its emphasis on shared responsibility and accountability among community members. Interventions should be developed with and led by community members to ensure their acceptability and longevity [51]. Our study captures the perspectives of men who align with their communities’ normative expectations during their spouse’s pregnancy. Future research can explore the community’s responses to men who do not fulfil their spousal and paternal obligations related to maternal health. Future research can explore how negofeminism in African contexts overturns male dominance in other aspects of women’s health.

## Conclusion

This paper makes an important contribution in presenting women’s influence and resistance by using negofeminism as a theoretical lens to explore maternal health in a rural Nigerian context. The authors acknowledge the complexities of theorizing maternal health including care-seeking and maternal wellness in a cross-cultural context and therefore highlight the importance of interrogating the source of the theory and its positionality through which a social, intellectual and political stance on maternal health is legitimized. Through conversations with men and women, this paper examines how discourses underlying negofeminism are tied to seeking maternal care and maternal health in general. Findings describe ways in which women were able to negotiate authority by ascribing the role of decision-maker to their men spouses while influencing their maternal healthcare decisions. Findings also reify negofeminism’s concepts of alliance, community and connectedness by highlighting men’s involvement in maternal health. Furthermore, women’s resistance to men’s involvement in their pregnancy was evidenced thereby challenging assumptions of rural women as passive and oppressed. The importance of incorporating inclusive approaches to involve women’s communities and spouses in maternal health programs cannot be overemphasized.

Despite advances in maternal health, there is much to understand regarding women’s health in African contexts. An African feminist approach deepens understanding of processes women navigate and how women are impacted. This approach allows for exploring women’s lives beyond pregnancy or childbirth and can also be used to understand women’s health throughout the life course. Highlighting women’s experiences through African feminism moves away from notions of deficit in women’s agency towards a recognition of the structural and institutional influences that can improve maternal health.

## Data Availability

The datasets generated and/or analyzed during the current study are not publicly available due to analysis being underway for subsequent publications. They are available from the corresponding author on reasonable request.
